# Clinical and radiological outcomes of total hip arthroplasty in patients affected by Paget’s disease: a combined registry and single-institution retrospective observational study

**DOI:** 10.1186/s10195-021-00574-y

**Published:** 2021-03-17

**Authors:** Alberto Di Martino, Maria Antonietta Rita Coppola, Barbara Bordini, Niccolò Stefanini, Giuseppe Geraci, Federico Pilla, Francesco Traina, Cesare Faldini

**Affiliations:** 1 1st Orthopaedic and Traumatologic Clinic, IRCCS Istituto Ortopedico Rizzoli, Via Pupilli, 1, 40136 Bologna, Italy; 2 Department of Biomedical and Neuromotor Science–DIBINEM, University of Bologna, Bologna, Italy; 3 Laboratorio Di Tecnologia Medica, IRCCS Istituto Ortopedico Rizzoli, Via di Barbiano, 1/10, 40136 Bologna, Italy; 4 Orthopaedic-Traumatology and Prosthetic Surgery and Revisions of Hip and Knee Implants, IRCCS Istituto Ortopedico Rizzoli, Via Pupilli, 1, 40136 Bologna, Italy; 5 University of Messina, Messina, Italy

**Keywords:** Paget’s disease, Total hip arthroplasty, Revision, Heterotopic ossification, Outcomes, Blood transfusion

## Abstract

**Background:**

Total hip arthroplasty (THA) in patients with Paget’s disease can be associated with technical difficulties related to deformities and altered mechanical bone properties, and hypervascularity leads to significative intra-operative bleeding. The purpose of this registry and single-institution study was to investigate overall survival and causes of failure of THA in pagetic patients, together with an analysis of the clinical and radiological complications.

**Material and methods:**

Registry-based survival and complication analysis, type of fixation, intra- and post-operative complications, clinical (pharmacological history, blood transfusions, Harris hip score [HHS]) and radiographic (cup orientation, stem axial alignment, osteolysis around the cup and the stem and heterotopic ossification [HO]) data were reviewed.

**Results:**

In total, 66 patients (27 males and 39 females, mean age at surgery 71.1 years for males and 74.8 years for female) from the registry study presented a 10-year survival of 89.5%. In the institutional study, involving 26 patients (14 males and 12 females, 69 years average) and 29 THAs, hip function improved significantly. Average cup orientation was 40.5°, while varus stem alignment was 13.8%. In total, 52% of hips had heterotopic ossifications. Peri-acetabular osteolysis was in 13.8% of implants and in 45% of hips was found around the stem. Allogenic and autologous blood transfusion rate were 68.2% and 31.8%, respectively, with an average transfusion of 2 units of blood (range 1–6 units). HHS improved by an average of 34 points, with excellent result in 64.3% of patients. Two implants failed, one due to traumatic ceramic head fracture 64 months after surgery, and one due to mobilization of the cup on the second post-operative day.

**Conclusion:**

THA surgery in Paget’s patients is a safe procedure, and implant survival is only partly affected by bone remodelling and choice of fixation. The post-operative functional outcome is largely similar to that of other patients. Bleeding-related complications are the main complications; a careful pharmacological strategy should be recommended to decrease the risk of transfusions and of HO development.

**Level of evidence:**

Level III

## Introduction

Paget’s disease of bone was described by Sir James Paget in 1877 [1]. It affects the axial skeleton asymmetrically, primarily involving the pelvis (60.3%), lumbar spine (35.1%), femur (32.3%), skull (22.2%) and tibia (15.5%) [2]. The disease is rare before 40–50 years of age, and the prevalence is about 1% in the population aged > 50 years, reaching 5% after 80 years [3]. Bone architecture in Paget’s disease is completely subverted by a disorganized bone tissue with single or multiple bone alterations, and monostotic and polyostotic forms have been described [4]. The disease is characterized by an increased number and activity of osteoclasts and activity of osteoblasts [5], leading to the formation of a hyperplastic bone which is mechanically weaker, less compact and more susceptible to fractures compared with a normal adult bone [6]. At the same time, bone marrow fibrosis and increased bone marrow vascularization are observed, particularly in the osteolytic and mixed phases of the disease [7–9], while in the sclerotic phase the osteoblastic activity predominates and a sclerotic, hypovascular bone is found [2]. Serum alkaline phosphatase derives from osteoblasts during the process of bone formation, whereas hydroxyproline is excreted in urine because of bone collagen disruption in the osteolytic phase [10]. Elevated serum alkaline phosphatase level is the main biochemical marker, since its increase is related to a metabolically active pathology and greater skeletal involvement [11], while hydroxyproline, although very sensitive, presents technical difficulties in collection and measurement [10].

Not infrequently, diagnosis is made when skeletal deformities or complications appear [2]; advanced stage Paget’s disease at the hip typically shows coxa vara, acetabular protrusio and anterolateral femoral bowing [12]. Anterolateral femoral deformity may cause disability because of deviation from the normal mechanical axis; therefore, corrective osteotomy could be useful to re-establish alignment of the joint. In acetabular protrusion, medial acetabular bone grafting, antiprotrusio cage and oversized hemispherical cup have been reported to restore hip centre of rotation. Sclerotic bone on the femoral side must be treated with caution, and a high-speed burr is recommended in bone preparation. To prevent varus positioning of the stem due to coxa vara in pagetic patients, osteotomies may be necessary [13]. Secondary arthritis of the hip in Paget's disease is the main complication since the deformity at the acetabular and femoral bones alters the mechanical loads, causing early joint degeneration [1] and requiring a joint replacement surgery if symptomatic.

Challenges related to surgery are due to the presence of bone sclerosis and deformities, the increased risk of peri-prosthetic fractures and the developing HO [6]. Implant fixation to the bone has been a matter for debate; bone cement has been widely used in the past, although some studies found a higher incidence of radiolucency at the bone–cement interface, and some authors support the use of uncemented implants [1, 12, 14, 15]. At present, only one registry study is available on the topic, reporting about 114 patients operated on for THAs [16], but like in many registry studies, clinical outcomes are missing.

This study therefore aims to evaluate overall prosthetic implants survival of THA through a regional registry evaluation; moreover, a medium-to-long-term clinical outcomes and radiographic evaluation of patients who underwent operation for THA at the authors’ institution has been performed.

## Materials and methods

The Central Emilia Wide Area Ethical Committee of the Emilia-Romagna Region (CE-AVEC) approved the study (Study HA-PAGET, protocol 0003593, March 5, 2020).

The register of orthopaedic prosthetic implants (RIPO) of the Emilia Romagna (ER), a region of North-East Italy, was asked to select patients affected by secondary arthritis secondary to Paget’s disease. RIPO reports data about hip, knee and shoulder arthroplasty procedures performed within the region. Founded in 1990, RIPO has a capture rate of approximately 95% on the implants performed in all orthopaedic departments of the region (both public and private), involving a total of 62 private and public hospitals. The design of this register was conceived to allow comparison with the most important national registries [17].

RIPO’s data include the following information: age, gender and clinical history of the patient; diagnosis leading to replacement; model and design of the implant; surgeon performing the procedure and in which hospital. When revision surgery is performed, it is captured by RIPO even in the case of surgery performed outside ER; in fact, any surgical procedure performed in any part of Italy is notified and billed back to the region of residence of the patient. Primary endpoint of the register is revision of one or more prosthetic implant components.

The diagnosis of Paget’s disease was provided by the surgeon filling in the RIPO form. The RIPO database was enquired about age, sex and kind of fixation of implants and overall survival when THAs were performed for secondary arthritis due to Paget’s disease between January 2000 and December 2017 in ER. Sample size has not been calculated since all the cases available from the RIPO registry were included and analysed.

No information is collected in the RIPO registry about clinical and radiological outcomes. To overcome this limit and outline the peri-operative course, Paget’s patients undergoing surgery for THA at the 1st Orthopaedic and Traumatologic Clinic or at the Orthopaedic-Traumatology and Prosthetic surgery and revisions of hip and knee implants, at IRCCS-Istituto Ortopedico Rizzoli, were selected, independently from the region of origin. From December 1999 to April 2019, 29 THAs were performed on 26 patients. Data of bisphosphonate therapy, type of implant, type of fixation, functional outcomes, intra- and early post-operative complications and re-operations were extracted. Variation of pre- and post-operative haemoglobin (Hb) level was analysed to determine the presence of anaemia (defined as Hb < 13 g/dL in males and < 12 g/dL in females) [18], and the number of autologous and allogenic blood transfusions was recorded. Paget’s disease activity at the time of surgery was assessed on the basis of pre-operative alkaline phosphatase levels, when available. Outpatient reports and radiographic images analysis, and hip prosthesis revision surgery data from RIPO registry were collected, when available. Functional outcomes were evaluated through the HHS at the latest clinical follow-up.

### Radiographic analysis

The pre-operative and post-operative leg length discrepancy (LLD) was obtained by measuring the vertical distance between the most prominent medial point of the lesser trochanter and the inter-teardrop line on standing X-rays [19]. HO was established on post-operative anteroposterior view according to Brooker classification [20].

Post-operative anteroposterior X-rays were used to assess cup and stem orientation, while the latest radiographic follow-up was analysed for the osteolysis around cup and stem. Cup orientation was assessed as the angle between the edge of the cup and the horizontal line of the pelvis [21]. The Lewinnek ‘safe zone’ with a cup inclination of 40° ± 10° was used as safe margin for cup orientation minimizing the risk of dislocation after hip arthroplasty [22]. Stem axial alignment in THA was carried out considering the angle between the stem axis and the proximal femoral axis, classified as varus–valgus tilt if it deviated by more than 5° from the femoral shaft [23].

Osteolysis around the cup was analysed by assessing the presence of continuous radiolucent lines of more than 2 mm in diameter at the bone–prosthesis interface in zones 1 to 3 according to DeLee and Charnley [24]. Cup loosening was defined as displacement of the cup by more than 2 mm or 5° [25]. Osteolysis around the stem was graded into seven zones according to Gruen et al. [26], assessing stem failure if there was 2 mm migration or varus–valgus tilting [25].

All radiographic measurements were obtained using Carestream Health Inc., the picture archiving and communication system (PACS) available at our institution.

### Statistical analysis

Statistical analyses were performed using SPSS 14.0, version 14.0.1 (SPSS Inc, Chicago, IL) and JMP, version 12.0.1 (SAS Institute Inc, Cary, NC, 1989–2007). Descriptive statistics were used to summarize the data, presented as median and mean with standard deviation (SD) for continuous variables and as frequency with percentage (%) for categorical variables. Statistical significance was calculated using the Mann–Whitney test for clinical quantitative data. A *p* value of < 0.05 was considered statistically significant**.** Survival curves were calculated and plotted using the Kaplan–Meier method. Prosthesis failure is defined as the revision of even one prosthetic component. Cox proportional hazards model was used to investigate the association between the survival time of implants and multiple predictive variables. Implants were followed until the last date of observation (date of death or date of visit).

## Results

The two different populations collected from registry report and single-institution clinical charts are compared in Table 1

**Table 1:** Comparison between populations collected from registry report and single institution

Population	Registry report	Single institution
Number	66 (27 M; 39 F)	26 (14 M; 12 F) 29 hips
Mean age at surgery, years	71.1 M; 74.8 F	69
Type of fixation		
Cementless	44/66	20/29
Cemented	9/66	1/29
Hybrid	13/66	8/29
Coupling		
Metal-on-polyethylene	23/66	1/29
Ceramic-on-polyethylene	18/66	3/29
Ceramic-on-ceramic	19/66	25/29
Metal-on-metal	2/66	–
Oxinium-on-polyethylene	2/66	–
Failures	8/66	2/29
Aseptic loosening of the stem	4/66	–
Aseptic loosening of the cup	1/66	1/29
Recurrent prosthesis dislocation	1/66	–
Breakage of head	1/66	1/29
Peri-prosthetic fracture	–	–
Unknown	1/66	–

### Registry report on implant survival in Paget’s patients

In ER, 106 Paget’s patients underwent THA surgery in the time interval between 1 January 2000 and 31 December 2017. Of these, 66 patients reside in ER, and data of these patients were used for the determination of implant survival and complications analysis. These 66 implants were inserted in the period between 1 January 2000 and 31 December 2017.

**Fig. 1:**
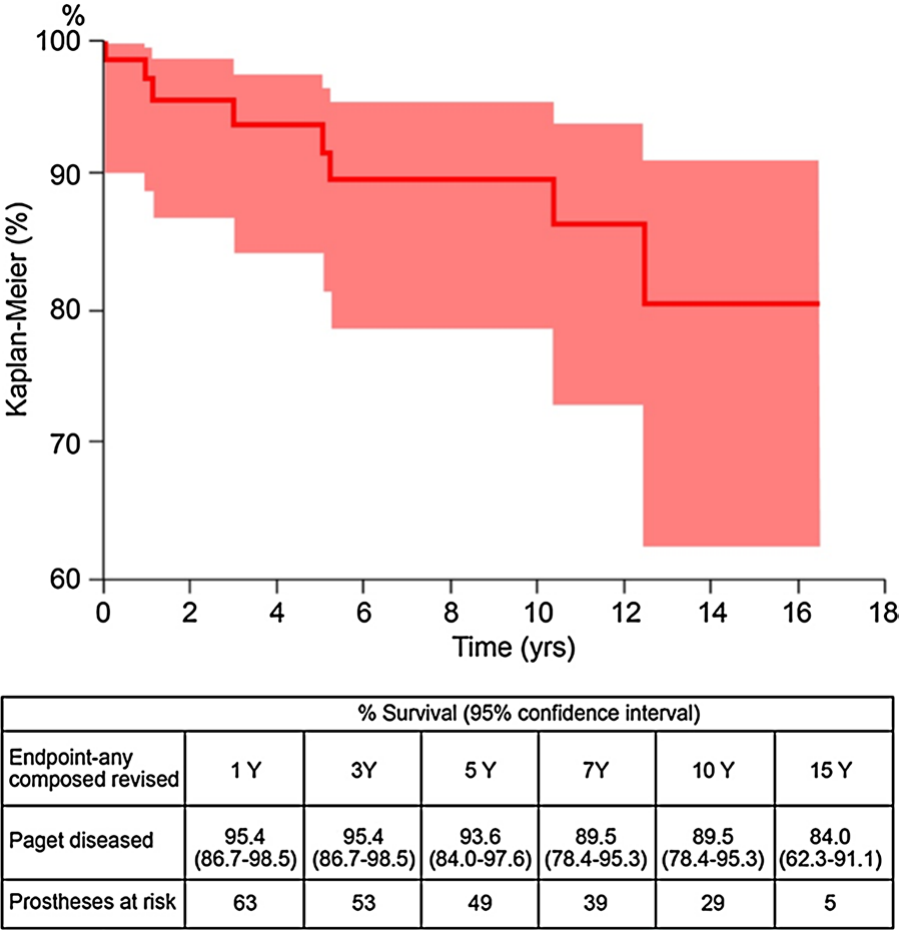
Survival of THA implants in Paget’s patients

Of the 66 patients, 27 were male and 39 were female. Mean age at surgery was 71.1 years for males (range 57–84 years) and 74.8 years for females (range 61–88 years). In total, 44 out of 66 implants (66.7%) were cementless, 9 were cemented (13.6%) and 13 were hybrid (19.7%). Articular coupling was metal-on-polyethylene in 23 patients (34.9%), ceramic-on-polyethylene in 18 patients (27.3%), ceramic-on-ceramic in 19 patients (28.8%), metal-on-metal in 2 patients (3.03%) and Oxinium-on-polyethylene in 2 patients (3.03%). In two patients, the report did not give data about articular coupling. Failures were recorded up to Dec 31, 2018. At an average follow-up of 8.4 years (range 0–16.6 years), eight patients underwent revision surgery, giving a 10-year survival of 89.5% (Fig. 1).

Aseptic loosening at the cup or stem were the main causes of revision, accounting for 62.5% of failures in the cohort (Table 2). When adjusted for gender, age and type of fixation, no influence was found on the outcomes of prosthetic surgery.

**Table 2:** Cause of revision in Paget’s patients, report from the RIPO registry

Cause of revision	Rate	Percentage	% Distribution failure causes
Aseptic loosening of the stem	4/66	6.1%	50.0%
Aseptic loosening of the cup	1/66	1.5%	12.5%
Recurrent prosthesis dislocation	1/66	1.5%	12.5%
Breakage of head	1/66	1.5%	12.5%
Unknown	1/66	1.5%	12.5%
Total	8/66	12.1%	100.0%

### Single-institution patient population and implant characteristics

#### Study population

At the authors’ institution, surgery involved 26 patients with 29 THA implants, 14 males (54%) and 12 females (46%), with 14 right hips (48%) and 15 left hips (52%). The average clinical and radiographic follow-up were 84.2 months (range 1–195 months) and 30.7 months (range 1–132 months), respectively. Twelve patients died, and three were lost during the follow-up. At the time of surgery, the mean patient age was 69 years (range 53–85 years) and the average body mass index (BMI) was 26.7 (range 23–33). There were 13 (45%) patients on bisphosphonate therapy before surgery. The mean operative time was 92.8 min (range 79–120 min), and the average hospital length of stay was 8.7 days (range 4–22 days). Twenty implants were cementless (69%), and 3/20 patients (15%) required cup fixation augmented by screws; in all cases, a two-hole cup was used and the two screws were oriented in the posterior-superior quadrant. Therefore, six screws were applied. Bone cement was used in nine hips, with one full cemented implant (3.4%), and eight (27.6%) patients had a cemented stem and a cementless cup (hybrid). In three hybrid implants (37.5%), two screws were added to improve cup stability. Used implants are presented in Table 3. The articular coupling was ceramic-on-ceramic in 25 hips (86.2%), ceramic-on-polyethylene in 3 hips (10.3%) and metal-on-polyethylene in 1 hip (3.5%).

**Table 3:** Choice of implant design, fixation

Cementless cup	*N*	Percentage, %	Screws
FIXA TI-POR (Adler Ortho S.P.A., Cormano (MI), Italy)	18	62.0	4
AnCA FIT (Wright Medical Group N.V., Memphis, Tennessee, USA)	4	13.8	1
TRIDENT (Howmedica Osteonics Corp., Mahwah, New Jersey, USA)	2	6.9	0
Duofit (Samo International, Granarolo dell’Emilia, (BO), Italy)	3	10.3	1
Mpact Medacta (Medacta International, Castel San Pietro, Switzerland)	1	3.5	0
Total	28	96.5	6

Cemented cup	*N*	Percentage, %	Screws
TRIDENT (Howmedica Osteonics Corp., Mahwah, New Jersey, USA)	1	3.5	0

Cementless stem	*N*	Percentage, %	
APTA (Adler Ortho S.P.A., Cormano (MI), Italy)	14	48.3	
AnCa Fit Wright (Wright Medical Group N.V., Memphis, Tennessee, USA)	4	13.8	
Exter (Howmedica Osteonics Corp., Mahwah, New Jersey, USA)	1	3.4	
Ami Stem-H (Medacta International, Castel San Pietro, Switzerland)	1	3.4	
Total	20	68.9	

Cemented stem	*N*	Percentage, %	
APTA (Adler Ortho S.P.A., Cormano (MI), Italy)	4	13.8	
Exter (Howmedica Osteonics Corp., Mahwah, New Jersey, USA)	2	7.0	
LC (Samo International, Granarolo dell’Emilia, (BO), Italy)	3	10.3	
Total	9	31.1	

#### Clinical and surgical outcomes

Fourteen HHS questionnaires were administered post-operatively at an average of 109.4 months (range 5–195 months). The missing 15 HHS are due to the inability to administer the survey because of the death of patients or patients not filling out the questionnaire. The HHS improved by an average of 34 points (*p* < 0.001) from an average pre-operative score of 55.2 points (range 16.9–92.7 points) to a post-operative score of 89 points (range 70.8–99.8 points). The overall HHS was excellent for 64.3% of patients.

Pre-operatively, patients showed Hb levels of 12.8 (range 9.4–16.6; median 12.9 and SD 1.6) g/dL, dropping to 9.4  (range 7.3–13.2; median 9.25 and SD 1.37) g/dL post-operatively. In the peri-operative period, 28 patients (97%) developed anaemia; in two cases (6.9%), presence of local hematomas was reported. In total, 22 patients (75.9%) received blood transfusions, with patients receiving an average of 2 units of blood (range 1–6 units). Allogenic and autologous blood transfusion rates were 68.2% and 31.8%, respectively.

In the single-institution cohort, THA survival rate was 96.6% (79.2–99.5%) at 5 years with 15 prostheses at risk, and 89.1% (62.9–97.5%) at 7 years with 11 prostheses at risk. THA revision rate was 6.9% at 7 years. There was no clinically significant difference between high pre-operative alkaline phosphatase levels in patients who had implant failure and those with 7-year implant survival. At the time of clinical follow-up, two implants have been revised. The first patient was an 81-year-old man who sustained a ceramic head fracture 64 months after surgery for a femoral neck fracture (Fig. 1).

In the second patient, a proximal femoral fracture was synthetized intra-operatively by a metallic cerclage wire, and on the second post-operative day the patient exhibited mobilization of the cup, requiring reoperation by implant of a revision cup stabilized by screws; the patient developed hip dislocation managed by closed reduction.

### Radiographic results

The pre-operative LLD averaged −3.4 mm (−18 to +20), while post-operatively it was +3.2 mm (−30 to +24). In 19 patients (65.5%) post-operatively, a limb length discrepancy less than or equal to 10 mm was found. Average cup orientation was 40.5° (range 18.8–52.9°), and there was only one cup (3.4%) with an inclination angle < 30°. In total, 13.8% of stems were in varus with an average of 1.4° of varus (range 0.89–1.93°), whereas no stem was aligned in valgus position. HO at an average 2 years after surgery was found in 15 hips (51.7%) and, specifically, following the Brooker classification, six hips (40%) were found in class 1, six hips (40%) in class 2 and three hips (20%) in class 3 of HO.

**Fig. 2:**
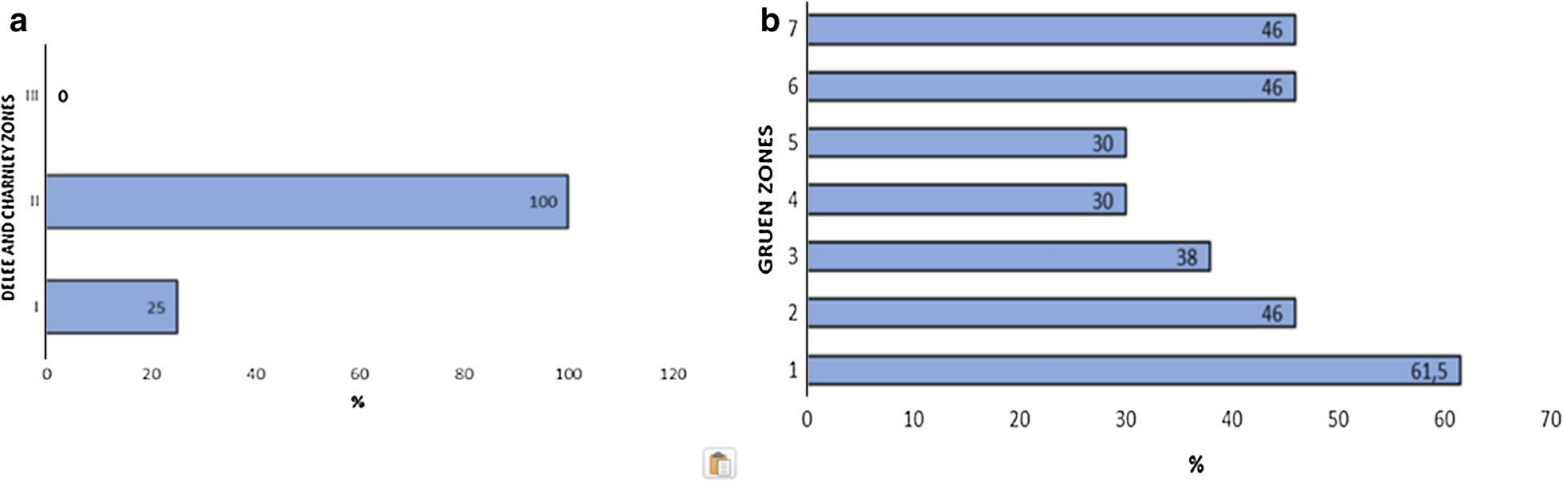
Bar chart showing the percentage of peri-prosthetic osteolysis according to DeLee and Charnley (**a**) and Gruen (**b**) classifications

Signs of peri-acetabular osteolysis according to DeLee and Charnley were noted in four implants (13.8%), affecting zone 2 in all four cases and zone 1 in one case; osteolysis around the stem mainly affected Gruen’s zone 1 (61.5%), followed by zones 2, 6 and 7 (46%) (Fig. 2).

**Fig. 3:**
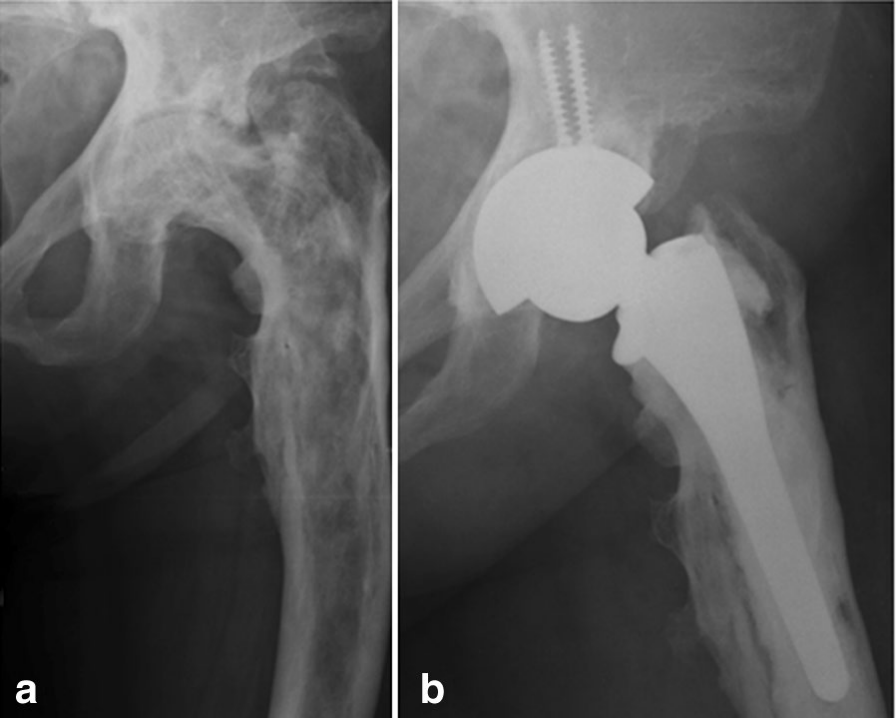
**a** Pre-operative anteroposterior X-ray of the hip of a woman who was 60 years old at the time of surgery; **b** 1-year post-operative X-ray showing wide radiolucent lines along the cement–bone interface at the stem

Radiolucent zones around the stem were present in 13 hips (45%), with 7/9 (77.8%) cemented stems showing radiolucency at the cement–bone interface (Fig. 3).

## Discussion

In the current study, RIPO regional registry analysis showed that THA implants in patients operated on for secondary arthritis due to Paget’s disease had a 10-year survival of 89.5%. Aseptic loosening at the cup or at the stem were the main causes of revision, accounting for 62.5% of failures in the cohort (Table 1). When analysing the cohort of patients operated on at the authors’ institution, at an average 84-month follow-up, just two implant failures and a good improvement in functional outcomes were reported. Our finding on HHS improvement is in line with other studies; Imbuldeniya et al. [27] in a 12.3-year follow-up study reported an increased functional outcome, from 56/100 points pre-operatively to 83/100 points post-operatively. Similar results were described recently by the study of Tibbo et al. [28], in which mean HHS improved from 49 to 76 points. Peri-operative bleeding and HO represented the most frequent surgical complications in this patient population.

The overall survival rate, slightly lower when compared with that described in literature for non-pagetic patients undergoing the same surgery (96.7% [96.5–96.%] at 5 years and 94.0% [93.8–94.3%] at 10 years) [17], is in line with most studies on pagetic patients [29, 30]. Our registry results regarding complications suggest that aseptic loosening is the main reason for implant failure requiring revision, accounting for 6/8 revision surgeries, and are in line with other authors’ findings that revision rates ranged 0–9% at mid-term [16, 28, 31, 32]. Tibbo et al. [28] reported the results of 17 primary THAs in patients with Paget’s disease at 8-year follow-up; the cohort had one revision for aseptic loosening of a cemented femoral component. In a recent registry study by Makaram et al. [16], revision THA is reported in 2.8% of 144 pagetic patients, with a THA implant survival of 96.3% at 10 years, that is, higher than in our cohort or current available literature.

THA performance in the pagetic patient has long been considered a challenge because of the characteristic imbalance between bone resorption in the osteolytic phase and deposition during the osteoblastic phase that leads to skeletal deformities, structural weakness of bone and altered joint biomechanics [1]. All of these may compromise osseointegration, resulting in early implant failure [6]. However, when observing data from recent studies, implant malpositioning, intra-operative fractures and mechanical complications of THA implants are not frequently reported. In particular, in a study by Wegrzyn et al. [32], the femoral component was in a neutral position in 90% of hips, and stem in slight valgus in 8% and varus in 2% of cases. Makaram et al. [16] reported that the most common surgical complications within 1 year of primary THA were haematoma formation (1.4%) and dislocation of prosthetic joint (1.4%).

**Fig. 4:**
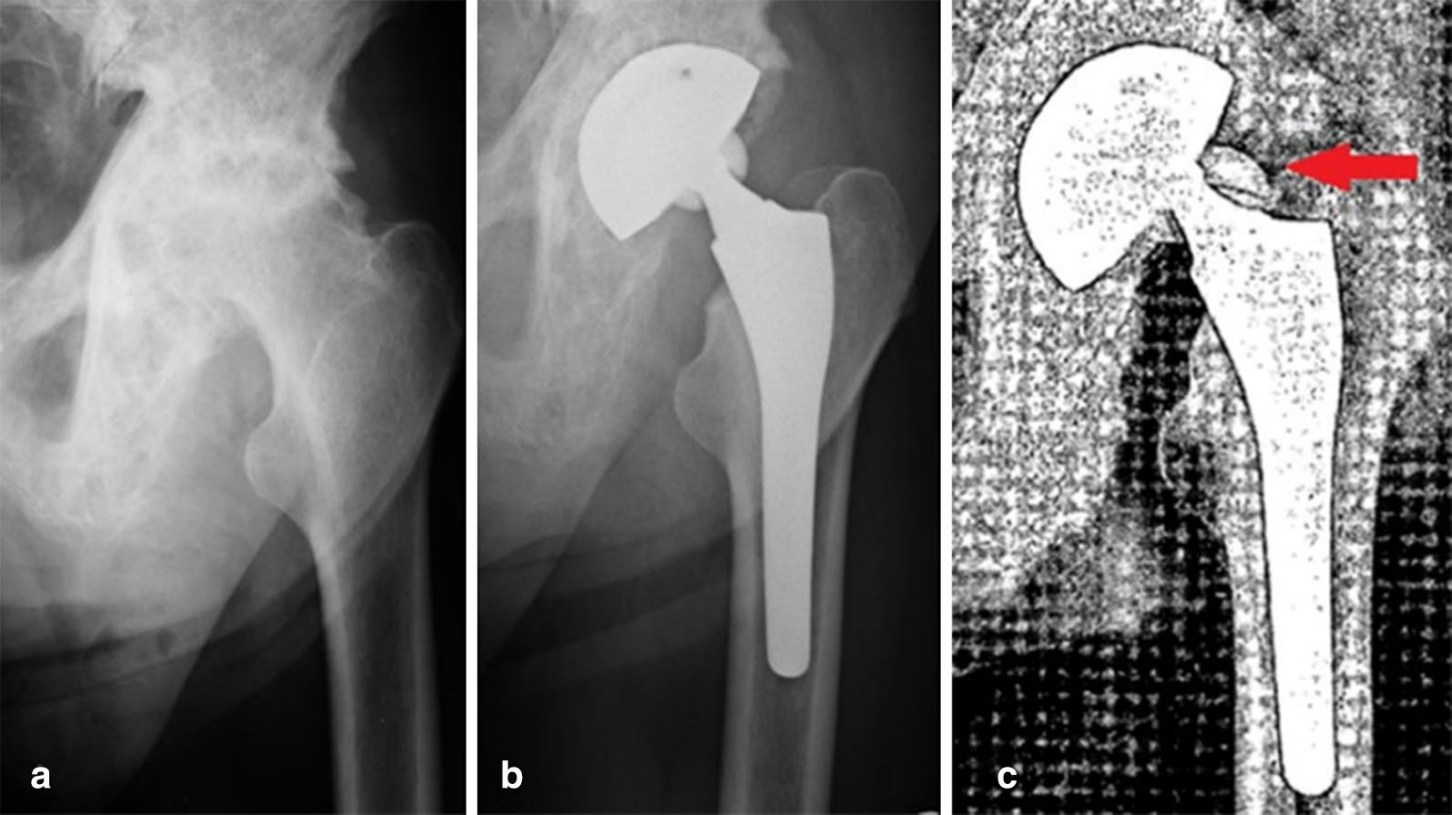
**a** Pre-operative anteroposterior X-ray of the left hip of a man who was 76 years old at the time of surgery, showing arthrosis secondary to Paget’s disease affecting the upper femur; **b** post-operative radiograph: cementless THA; **c** anteroposterior view of the pelvis after traumatic ceramic head fracture occurred 64 months after surgery

In our institutional cohort, we reported one intra-operative femur fracture managed by wiring, four varus stem positionings and one cup positioning out of Lewinnek safe zone (that was associated with an early post-operative dislocation and required cup revision). Horizontal cup placement (3.4% of hips) and varus stem positioning (13.8% of hips) were possibly due to the bone deformities of pagetic patients [12]. Several studies have shown that coxa vara and femoral bowing, characteristic of advanced stages of Paget’s disease, can complicate the entry of the stem into the femoral canal, increasing the risk of varus implant alignment. One patient was reoperated on because of ceramic head fracture (Fig. 4). In literature, the fracture rate of ceramic components has been reported to be between 0.013% and 1.1% of patients undergoing ceramic-on-ceramic THA and can be associated with altered local coupling and implant biomechanics [33].

Radiolucency around implants in pagetic patients has long been a debated issue. Consistent with the results of other studies [32], we reported a 13.8% incidence of peri-acetabular radiolucency and a 77.8% incidence of cemented stem osteolysis, as assessed by the presence of fractured bone cement. For this reason, the type of fixation has long been considered a crucial issue in Paget’s patients. In the past, a lot of cemented implants were used in these patients, to overcome the potential issue of poor bone quality and to improve fixation [14, 30]. However, bone cement can be challenging in these patients because of bone sclerosis contrasting cement penetration in the trabeculae, and also because of bone remodelling that could weaken the bone–cement interface [1]; for these reasons, the use of uncemented implants has been advocated [1, 6]. In general, both cemented and uncemented implant revision and survival rates are similar [6, 15]. In a recent review by Hanna et al. [6], no difference in revision rates between cemented and uncemented implants was found. In our patient population, no stem and one cup loosening requiring revision were registered at 31-month radiological and 84-month clinical follow-up.

Bone hypervascularization in Paget’s patients, which is related to the inability to produce a dry bed for cement interdigitation, may compromise short-term and mid-term implant fixation [12]. Moreover, peri-operative bleeding and HO were the main complication encountered in the institutional study. An average variation in Hb levels of 3.4 g/dL after hip surgery and a frequent rate of blood transfusions (75.9%) has emerged from the current study, significantly higher than results available in literature [34] in non-pagetic THA patients, highlighting the use of greater amount of blood transfusion in pagetic patients [1]. At present, pre-operative anti-pagetic therapy with intravenous bisphosphonates and peri-operative use of tranexamic acid are recommended in these patients to reduce intra- and post-operative bleeding [6, 35]. The observed incidence of HO in the institutional cohort was 52%, similar to previous published research [28, 29]. It is unclear how the surgical approach can affect HO incidence, and there are no unequivocal opinions concerning the best procedure to prevent HO in terms of radiation therapy (single 7–8 Gy dose) [36, 37] or chemoprophylaxis (including indomethacin, diclofenac or ibuprofen) [38–41].

Our cohort findings confirm the significative improvement in functional outcomes and pagetic patient satisfaction after hip replacement surgery, which shows an excellent clinical outcome in most cases, results which are widely comparable to the outcome of THAs in non-pagetic patients [6, 42]. THA surgery determined a significant pain resolution, improved joint function and quality of life, with good medium- and long-term implant survival even if slightly lower than in non-pagetic patients undergoing a primary THA.

Our study presents several limitations, for both the registry and the clinical study. In the registry study, it was not possible to assess the pre-operative conditions and the post-operative outcomes from clinical and radiological perspectives. Besides, RIPO collect cases from different hospitals and departments, in which different implant designs and even different surgical techniques are used; these could theoretically affect the results. However, one of the major strengths of registry research lies in its numerosity, which should overcome different instrumentations. Moreover, conservatively treated complications of the implants (with a special regard to dislocations) could not be captured. The single-institution report is limited by the retrospective design of the study and the high variability of follow-up intervals as well as the inability to verify surgical revisions in patients not residing in ER. However, our findings are in agreement with most available literature on the topic [6, 28].

In conclusion, THA surgery in Paget’s patients is a safe procedure when in experienced hands, and implant survival is only in part affected by bone remodelling and choice of fixation. Since bleeding-related complications still appear as the main issues, a careful pharmacological strategy should be recommended in the pre-operative setting of THA surgery to decrease the risk of transfusions and of HO development.

## Data Availability

Data collection is from RIPO register (Laboratorio di Tecnologia Medica, IRCCS Istituto Ortopedico Rizzoli) and from clinical charts (1st Orthopaedic and Traumatologic Clinic, Orthopaedic-Traumatology and Prosthetic surgery and revisions of hip and knee implants, IRCCS Istituto Ortopedico Rizzoli).

## Abbreviations

THA: Total hip arthroplasty
HHS: Harris hip score
RIPO: Register of orthopaedic prosthetic implants
ER: Emilia Romagna
Hb: Haemoglobin
LLD: Leg length discrepancy
PACS: Picture archiving and communication system
SD: Standard deviation
BMI: Body mass index
HO: Heterotopic ossification
